# eGenomics: Cataloguing Our Complete Genome Collection III

**DOI:** 10.1155/2007/47304

**Published:** 2007-04-30

**Authors:** Dawn Field, George Garrity, Tanya Gray, Jeremy Selengut, Peter Sterk, Nick Thomson, Tatiana Tatusova, Guy Cochrane, Frank Oliver Glöckner, Renzo Kottmann, Allyson L. Lister, Yoshio Tateno, Robert Vaughan

**Affiliations:** ^1^Molecular Evolution and Bioinformatics Section, Oxford Centre for Ecology and Hydrology, Mansfield Road, Oxford, Oxfordshire OX1 3SR, UK; ^2^Department of Microbiology and Molecular Genetics, Michigan State University, East Lansing, MI 48824, USA; ^3^The Institute for Genomic Research, 9712 Medical Center Drive, Rockville, MD 20850, USA; ^4^European Molecular Biology Laboratory Outstation–The European Bioinformatics Institute (EMBL-EBI), Wellcome Trust Genome Campus, Hinxton, Cambridge CB10 1SD, UK; ^5^The Pathogen Sequencing Unit, The Wellcome Trust Sanger Institute, Wellcome Trust Genome Campus, Hinxton, Cambridge CB10 1SA, UK; ^6^National Center for Biotechnology Information, National Library of Medicine, National Institutes of Health, 8600 Rockville Pike, Bethesda, MD 20894, USA; ^7^Microbial Genomics Group, Max Planck Institute for Marine Microbiology and International University Bremen, 28359 Bremen, Germany; ^8^CISBAN and School of Computing Science, Newcastle University, Newcastle upon Tyne NE1 7RU, UK; ^9^Center for Information Biology and DNA Data Bank of Japan, National Institute of Genetics, Research Organization of Information and Systems, Shizuoka 441-8540, Japan

## Abstract

This meeting report summarizes the proceedings of the “eGenomics: Cataloguing our Complete Genome Collection III” workshop held September 11–13, 2006, at the National Institute for Environmental *e*Science (NIE*e*S), Cambridge, United Kingdom. This 3rd workshop of the Genomic Standards Consortium was divided into two parts. The first half of the three-day workshop was dedicated to reviewing the genomic diversity of our current and future genome and metagenome collection, and exploring linkages to a series of existing projects through formal presentations. The second half was dedicated to strategic discussions. Outcomes of the workshop include a revised “Minimum Information about a Genome Sequence” (MIGS) specification (v1.1), consensus on a variety of features to be added to the Genome Catalogue (GCat), agreement by several researchers to adopt MIGS for imminent genome publications, and an agreement by the EBI and NCBI to input their genome collections into GCat for the purpose of quantifying the amount of optional data already available (e.g., for geographic location coordinates) and working towards a single, global list of all public genomes and metagenomes.

## 1. INTRODUCTION

The Genomic Standards Consortium (GSC) is an initiative working towards richer descriptions of our collection of genomes and metagenomes (further information about Genomic Standards Consortium can be found at http://gensc.sf.net) [1]. Established in September 2005, the goal of this international community is to promote mechanisms standardizing the description of genomes and the exchange and integration of genomic data. Genomic sequencing projects are being completed at a rapid pace that will only increase as the application of ultra-high-throughput methods becomes commonplace. The primary aim of developing a new genomic standard is to ensure that those researchers generating genomes contribute to an increase in the quality and quantity of metadata, so that interpretation and analyses of the genome collection can be carried out in a comprehensive and unhindered manner, especially from an ecological and environmental perspective [2]. More background information about the GSC can be found at its website http://gensc.sf.net.

The 3rd workshop was organized by Dawn Field (Oxford Centre for Ecology and Hydrology) and Tatiana Tatusova (National Center for Biotechnology Information) and took place at the National Institute for Environmental *e*Science (NIE*e*S) in Cambridge, England, on 11–13 September, 2006. Participants included developers of community-based standards, computer scientists, researchers building genomic databases and conducting large-scale comparative genomic analyses, and biologists from various disciplines who are applying genomic data in their own settings. These participants included representatives of major sequence databases (DDBJ/EMBL/NCBI) and sequencing centres (JGI/Sanger/TIGR), a combination which proved essential for building the future roadmap for the GSC. The workshop built upon the previous two workshops, the first of which [1] led to the formation of the GSC and the second of which [3] aided its integration with the wider “OMICS” standardization community.

The workshop began with an introduction from the organizers. Dawn Field (Oxford Centre for Ecology and Hydrology) welcomed returning and new participants and emphasized the need to place GSC activities within the context of wider international standardization activities, many of which were represented by speakers at this meeting. Tatiana Tatusova (NCBI Entrez Genomes) further set the context for the event by relating her memories of the phenomenal growth in the number of genomes over the past 10 years. She also underscored the need to work with the wider community, highlighting the recent ASM/NCBI Workshop on Microbial Genome Annotation, Washington, DC, USA and the National Academy of Sciences study of metagenomics as two examples of recent allied initiatives.

## 2. SESSION I: OVERVIEW OF OUR CURRENT AND FUTURE GENOME COLLECTION

As Dave Ussery (Technical University Denmark), the session chair, stated in his introduction, the first session was designed to “remind everyone of the problem.” Sandie Baldauf (University of York) kicked off the meeting with an overview of eukaryotic diversity by reviewing current understanding of the eukaryotic tree of life [4]. For each of the eight major lineages of eukaryotes, she described the salient features of representative species and presented an estimate of the number of finished and future genomes that would be available for specific taxa. To date, animals and fungi remain the best sampled taxa by far, while the majority of eukaryotic lineages are represented only by an EST project, or not at all. Single-celled eukaryotes were highlighted as fascinating, not only because of their unusual molecular biology (e.g., ciliates have massively scrambled genes, euglenoid plastid genomes have twintrons (introns within introns) and trypanosomes have massive RNA editing of mitochondrial transcripts), but also because of their intriguing biological features (e.g., dinoflagellates cause various types of toxic shellfish poisoning and produce the most potent toxins known to science). Eukaryotic diversity will remain under-sampled for the near future, but numbers of known species are expected to increase rapidly. Eukaryotic microbial genomics is only now beginning its exponential growth phase just as bacterial microbial genomics did 10 years ago. Difficulties arise in the selection of eukaryotic genomes as some, like many protists, have extraordinarily large genomes and include large quantities of repetitive DNA.

Rob Edwards (San Diego State University) started his talk with slides of a sampling trip to Christmas Island, explaining this was the reason he missed the first GSC workshop. Rob proceeded to describe a range of metagenomic data sets from a variety of environments that have been generated with 454 pyrosequencing technology. In total, Rob has collected information from 71 libraries (12 from collaborators), 2 of which were published, and 12 of which were in the INSDC databases at the time of the workshop. He remarked that while the rate of sequencing has increased tremendously, the average read length has not and is currently 103 bp. Short-read length and massive amounts of data continue to make the informatics of 454 data sets challenging. However, Rob showed several examples where such data is providing insights into the genes and functions of organisms from a range of habitats (see, e.g., [5]). With the growth in environmental metagenomics projects, he stressed the importance of including global positioning system (GPS) coordinates (latitude and longitude) for each sample. These are critical fields, already found in the MIGS specification and supported by the optional “/lat_lon” qualifier in INSDC files. Submission to public databases is in progress, and all datasets are available from http://scums.sdsu.edu/. For further reading, Rob has authored a white paper on random community genomics [6].

George Kowalchuk (Netherlands Institute of Ecology) discussed the Dutch Ecogenomics program (http://www.ecogenomics.nl) a cooperative effort of institutions and companies financed by natural gas tax revenues and overseen by the Netherlands Genomics Initiative (http://www.genomics.nl). Molecular methods in microbial ecology of soils are starting to answer the simple question of “what is there?” and it is proposed that integrated (meta-)genomics approaches will start to provide a greater understanding of the more important question of “what are they doing?” The major themes of the program are bioremediation, ecological insurance, ecotoxicology and disease suppression (health interactions), all of which are brought together via overarching bioinformatics and technological platforms. He stressed that there is a growing need to work on a “microbially relevant” scale to pick apart the biology of complex communities and to do so, his group is currently using targeted metagenomic approaches and sequencing of key taxa within characterized communities. Such studies will help characterize the normal operating range of life support functions via an integrated ecogenomics approach and the study of the interactions of internal and external stress factors.

Paul Gilna (University of California, San Diego) gave an introductory talk about a new project to build a community resource for microbial ecologists who use metagenomics to study natural diversity. The recently launched “Community Cyberinfrastructure for Advanced Marine Microbial Ecology Research and Analysis” project, or CAMERA, has a five-year grant of 24.5 m from the Moore Foundation to build the computational infrastructure required for large-scale analyses of metagenomic data sets, with special emphasis on the global ocean survey (GOS) samples from the Sorcerer II voyage. In addition to sequences, he pointed out that each sample site could be linked to vast quantities of other data, including terabytes of satellite data. Metadata that has been captured for the GOS samples includes information on the site, sampling, and experimental parameters (i.e., filter applied to separate different size organisms prior to sequencing or insert size). He stressed the need to learn from history and to remember that the growth of databases in the coming years is not linear. CAMERA intends to use next generation computational infrastructure including tiled-wall videoconferencing rooms, the lambda rail (10 GB network), and the TeraGrid (1000s of CPUs) to provide access to data for metagenomic researchers across the US and beyond.

The clear theme to emerge from the opening session was a sense of the vast number of genomes and metagenomes that will be available in the near future, the potential this technology offers to better understand the natural world, and the wide range of technological advances that will be derived from these efforts. There was a general feeling that the global genomics initiative was comparable to the space race of the 20th century and the overall social and economic benefits would be as great or greater. As such, it sets the stage for a further set of presentations on how the international community can ensure that this data can be dealt with and used at its full potential.

## 3. SESSION II: DATABASES AND METADATA CAPTURE AND EXCHANGE EFFORTS

George Garrity (Michigan State University) chaired the next session on international metadata capture and exchange efforts. Tatiana Tatusova (NCBI) spoke on the sequencing project registry and how information about genome sequencing projects will be exchanged between collaborators using a web services protocol. Persistent identifiers for genomes and genes are part of the essential infrastructure for the future organization of the complete genome collection [7]. After presenting on the annotation of the complete *E. coli* K-12 genome and genomic resources at DDBJ, Yoshio Tateno (DDBJ) spoke about the systematic evaluation and classification of the predicted proteins in the complete bacterial genomes in the INSDC. In the Gene Trek in Prokaryote Space (GTPS) project, proteins in the bacterial genomes have first been predicted using Glimmer and RBSfinder, and then evaluated and classified by BLASTP and InterPro into six grades. The predicted proteins have then been further compared with all genes in the bacterial division of the INSDC. The results of the comparison were also used for the evaluation and classification. Among all predicted proteins (1,254,150), 556,815 were evaluated as currently reliable ones. The methods and results of GTPS are presented at http://gtps.ddbj.nig.ac.jp.

Natalia Maltsev (Argonne National Laboratory) was present at the first GSC workshop but could not attend this meeting, therefore Dawn Field presented the Maltsev lab's new project to make genomic annotations freely available in GFF3 format. The repository can be found at ftp://ftp.mcs.anl.gov/pub/compbio/PUMA2/gff/gff_files. A reoccurring theme throughout the workshop was the strong desire of members of the GSC to see downstream analyses held in various databases seamlessly integrated with INSDC files to produce an integrated source of information about genomes. The group agreed that GFF3 was a viable approach that should be supported but that a significant amount of community consensus-building would have to precede such activities as it is possible to create GFF-compliant files that are not easily integrated because, for example, they use different sets of features or optional fields.

At the end of this session Peter Sterk (EBI), Dawn Field (CEH Oxford), and Tanya Gray (CEH Oxford) presented an overview of the current status of the MIGS specification, its implementation as an XML schema, and a demonstration of the alpha release of the Genome Catalogue (GCat) software. This introduction was aimed at providing a background for discussions on Day 2. Progress since the last two workshops has included the following.

- Launch of the GSC website: http://gensc.sf.net.
- The publication of a special issue of the journal *OMICS*: a journal of integrative biology [8], which included contributions by GSC members and the meeting report from the 2nd GSC workshop [3].
- The drafting of MIGS 1.0 checklist and implementation as an XML schema (with the initiation of suitable controlled vocabularies).
- Alpha release of the Genome Catalogue (GCat) software.

In brief, the current version of MIGS that emerged from the first two GSC workshops has now been implemented as an XML schema for the purpose of discussing the information to be captured. The GCat software has been developed to provide a web interface that is generated “on-the-fly” from an underlying XML schema. GCat is designed to have a low development overhead which makes it especially useful in the short term while the MIGS specification is in flux. The benefit of this early implementation is that the GSC can support both the discussion of MIGS with case study genomes and the collection of MIGS-compliant genome reports. GCat has been developed in collaboration with the GSC implementation working group.

## 4. SESSION III: ALLIED PROJECTS AND ONTOLOGY DEVELOPMENT

On the second day of the workshop, the focus shifted to allied projects that are already leading the way in the area of the standardization and integration of biological information. Dawn Field chaired a session on a series of such projects, which had all been selected for their immediate relevance to the GSC and its future aims. George Garrity (Michigan State University) presented the NamesforLife (N4L) (http://www.names4life.com) project [9], a prototype of which is accessible via the DOI resolver (http://dx.doi.org/10.1601/tx.0). This prototype aims to disambiguate and future-proof biological nomenclature by combating the knowledge bleed that occurs when information dispersed in the scientific literature and databases is no longer accessible because key search terms (names) and concepts (taxa) have changed over time. The N4L technology is based on a semantic resolution service that couples Digital Object Identifiers (DOIs) with an ontology that expresses nomenclatural acts and taxonomic concepts as a collection of XML information objects that are persistently addressable and resolve nomenclatural acts in a contemporaneous manner. A key benefit of using DOIs is the ease of integration with the published literature, databases, and other electronic sources of information that have already adopted this standard.

George also spoke briefly about a related initiative, lead by Rick Stevens (Argonne National Laboratories) and Eddy Rubin (Joint Genome Institute) to produce draft genome sequences for all of the taxonomic-type strains of prokaryotes. The project would take approximately five years to complete, provide much needed reference genomes that are essential for correct assembly of metagenomes, fill existing phylogenetic gaps, and provide a foundation to meaningful linkage to vast amounts of data, information, and knowledge about these organisms that would significantly enhance inference. The obvious benefits of the proposal were immediately seized upon by participants.

Chris Taylor (European Bioinformatics Institute) described the new MIBBI project, an initiative aimed at bringing the MIxxx community of “Minimum Information” checklist developers together to create a unified source of “OMICS” checklists (http://mibbi.sf.net). The future goal of the project is to formulate an MIBBI Foundry in which participants will commit themselves to the integration of the ever-growing list of checklists such that the community can work towards multiomic standards. MIBBI has been driven by the Protein Standards Initiative (PSI), the Reporting Structures for Biological Investigations (RSBI), and the GSC. It represents a valuable opportunity for the GSC to work more closely with a wide range of standardization activities in the “OMICS” and allied sciences.

The next three talks focused on international efforts at ontology development. Michael Ashburner (University of Cambridge) gave the history of ontology work that has arisen from the development of the Gene Ontology (GO). From the beginning, one “problem” with GO was that it contained many other implicit ontologies—chemical compounds, for example. Over time, an increasing number of ontologies appeared, and GO developers became concerned that each was being developed independently. The GO developers essentially wanted a one-stop-shop, and established the Open Biomedical Ontologies (OBO) Library as a sourceforge site (http://obo.sf.net), encouraging colleagues to submit their ontologies to the collection. Now the number of registered ontologies has increased to more than fifty and OBO will be taken over by the recently founded NIH-funded National Center for Biomedical Ontologies (NCBO). Through this funding it will be possible to add more functions and services to OBO. Within a year, compound terms like “myoblast fusion” should be deconvoluted by explicitly referencing orthogonal ontologies (in this case the cell-type ontology). Finally, the OBO-Foundry is an effort to propagate best practices and to develop truly orthogonal ontologies.

Trish Whetzel (University of Pennsylvania) presented an overview of the ontology for biomedical investigations (OBI, previously known as the ontology for functional genomics investigation, or FuGO). OBI aims to provide an ontology for the unambiguous description of the components of biomedical (biological) investigations including the design, protocols and instrumentation, material, data, and types of analyses used. The application of this ontology to the annotation of a wide range of investigations would allow consistent annotation of data across technological and biological domains, thus enabling powerful concept-driven queries over the data. She presented an overview of which parts of the MIGS specification could be placed within OBI.

Additionally, phenotypic descriptions in MIGS could use the newly established phenotype and trait ontology (PATO), described by Suzi Lewis (Berkeley). The development of this ontology has been driven by the need to help the biomedical community describe the phenotypes associated with specific genes in different taxa, but is now becoming wider in scope due to interest from a variety of communities. Both OBI and PATO are part of the OBO Foundry, which aims to provide a unified set of ontologies that can explicitly describe organisms and their molecules, phenotypes, and traits. The day when the semantic resolution espoused by OBO becomes possible is drawing ever nearer.

The last speaker in the session was Frank Oliver Glöckner (Max Planck Institute for Marine Microbiology) who discussed the need to place sequences into their proper environmental context (e.g., marine, terrestrial, symbiotic). He suggested the exact location (GPS), depth (altitude), and time (x, y, z, t) of any sample be taken in any molecular field study. This geospatial information can then be used as a universal anchor to allow for sequence data in the context of prevailing biodiversity and habitat parameters. It will also allow supplementing the on-site information with dynamic data layers from global monitoring systems leading to an integrated ecosystem assessment.

He introduced the International Census of Marine Microbes (ICoMM) initiative (http://icomm.mbl.edu) as an additional source of geo-referenced data for microbial diversity, detailed the Metafunctions project (www.metafunctions.org) that integrates genomic information with habitat parameters, and described the design and use of the Megx.net database [10]. Furthermore, he introduced “Minimum Information about a Metagenomic Sequence” (MIMS) as an integrated extension of MIGS (http://gensc.sf.net). In addition to the core information captured in MIGS on latitude, longitude, depth (altitude), time, and date of sampling, MIMS would capture a more extensive list of habitat parameters that provide a rich set of contextual data for the sake of hypothesis generation and testing as well as ecosystems biology.

In the absence of Nikos Kyrpides (Joint Genome Institute), Dawn Field briefly demonstrated the Genomes Online Database (GOLD v2.0) [11], in particular pointing out the new search engine, the inclusion of the descriptors phenotype, ecotype, disease, project relevance and availability and their controlled vocabularies. Nikos reports that while many authors submit data directly to GOLD, he still curates a large amount of data and is keen to have community input. The group expressed interest in having these controlled vocabularies made available to the wider community. It also underscored the value of GOLD as an authoritative genomic database with an extensive user community that should be tightly integrated into any future GSC strategy.

## 5. SESSION IV: GROUP DISCUSSION OF THE MIGS SPECIFICATION, THE GENOME CATALOGUE, AND FUTURE TERM CAPTURE ACTIVITIES

This session marked the shift in the workshop from formal presentations to group discussion. All participants moved to a computer room where each had an access to a computer for the purpose of evaluating the GSC website and the Genome Catalogue. The session was led by Dawn Field and Jeremy Selengut (TIGR) and started with a discussion of the MIGS specification. In particular, the group focused on fields which were candidates for removal from the current specification, which helped the group to better define the general scope of the specification. As a result, the GSC agreed that all fields must meet the following criteria:

- to be an *appropriate extension* of existing INSDC qualifiers and information collected in the INSDC Project Metadata database;
- to consist of *objective facts* about genomic investigations (information that, ideally, the generators of a genome can best provide, but this does not exclude input by relevant experts);
- to contain *specific information* about the genome sequenced, while general information (e.g., about a species) should be held in authoritative databases;
- to include *clearly defined* pieces of information using values selected from controlled vocabularies.

It was clear from initial discussions that each part of the MIGS specification was of varying importance to each researcher. To get a good overview of the importance of each field in the specification, a lightning round vote was taken for all fields in the specification, and the number of votes recorded. It was found that a few clear cases could be made for dropping or compressing fields by using them as controlled vocabulary terms in other fields of a more general nature. A complete list of modifications used to produce MIGS v1.1 following this workshop can be found in the GSC Wiki under “MIGS Change Log” (http://gensc.sf.net).

The discussions then shifted to the issues surrounding the use of the Genome Catalogue by the GSC and the development of future content. Jeremy Selengut talked about how it would be possible, using an intelligent interface, to step users through the input of data far more easily. By presenting more general questions first, users could be guided by relevant, context-dependent input forms. For example, users who selected “draft” genome would then be prompted to fill in information for “estimated size of genome” while those who selected “complete” genome would not. Similarly, submitters of metagenomic data would not be burdened with questions only relevant to single-isolate studies and vice-versa.

Rob Edwards (SDSU) then presented an excellent case study for the GSC by relating his experiences with metadata capture for his collection of metagenomic libraries. This collection of data sets makes an excellent case study. His take-home message is that researchers cannot be expected to comply with standards of annotation that are presented *post hoc*. Rather, the best chance of gaining compliance is to have such standards at the start of experimental work. Rob also stressed that, as a potential adopter of such a system, he would not be willing to enter data two or more times. He emphasized the need for having no redundancy in the submission procedure developed for users (e.g., to INSDC and GCat), which would require a tight linkage between submission forms.

The group followed this with a discussion of the offer made prior to the workshop by EMBL participants Guy Cochrane and Bob Vaughan to enter the EMBL genomes into GCat for the purpose of generating useful content which might encourage authors to submit further information. Tatiana Tatusova (NCBI) offered to do the same for the NCBI genome collection. It was agreed that doing so would allow the GSC to quantify the amount of optional information (e.g., lat_lon) that is already available in INSDC fields and make it possible to work together towards a single, global list of genomes and metagenomes in the public domain.

Finally, the group briefly discussed the capture of terms in genome reports and agreed to continue work towards the posting of controlled vocabularies already in use by the community to the GSC website. All terms used to complete MIGS-compliant genome reports will be submitted by default to OBI [12] unless a more relevant ontology already exists.

## 6. DAY 3: ROADMAP AND WRAP-UP DISCUSSIONS

To start the day, Peter Sterk led a panel discussion with members of the INSDC. The INSDC was represented by Bob Vaughan and Guy Cochrane of EMBL, Tatiana Tatusova of the NCBI, and Yoshio Tateno of the DDBJ. The INSDC has a long history of describing nucleotide sequences and is now dedicating substantial efforts to building custom solutions for managing genomic data [7]. Guy Cochrane began the session by giving an introduction to the INSDC and outlined how the collaborators come together each May to hold an annual meeting in which formal proposals for changes to INSDC policy can be considered. It was agreed that Guy, through EMBL, would take forward an agenda item to present the MIGS specification at the May 2007 meeting (it had already been briefly introduced in May 2006) and report back to the GSC.

The main issue addressed in this panel session was that of the “MIGS-to-INSDC” mapping, which provides a defined way for information in MIGS to be formatted for inclusion in EMBL/DDBJ/Genbank documents. Developed by Bob and Guy and approved by the INSDC, this mapping places each MIGS field into the official INSDC feature table (see http://www.ebi.ac.uk/embl/WebFeat/index.html). The most frequently used optional qualifier in the mapping is /isolation_source. When many fields in MIGS go into a single qualifier, they will be written out as modifiers of feature qualifiers (e.g., /isolation_source=“altitude: 1500 M” or /note=“ploidy level: tetraploid”). It was further discussed that any field not already mapped into an INSDC qualifier could be placed into community-regulated structured comments (using the convention of tag-value pairs) by the submitters of the original sequences. Guy Cochrane also raised the issue of some MIGS fields becoming a formal part of the INSDC optional source qualifiers. He suggested that EMBL would take forward a proposal to add “health/disease status of host” to the next INSDC Collaborators meeting in May 2007.

## 7. THE GSC ROADMAP

The final session of the meeting was moderated by the GSC coordinators (George Garrity, Nick Thomson, Jeremy Selengut, Peter Sterk, Tatiana Tatusova, and Dawn Field). This session was dedicated to summarizing agreed action points and building consensus on the way forward. During the discussions on Day 2, Paul Gilna (UCSD) observed that the GSC is well placed to lead by example on the issue of adoption of MIGS. This fact came into focus on Day 3 when the GSC agreed that, as part of developing a presence in the genomics community, members would work to develop a logo, advertise the GSC website, advertise the GSC goals and aims in relevant public presentations, talk to their home institutions about adoption of MIGS, and request official permission to use the logos of participating projects and institutions on the GSC website. Perhaps most importantly GSC participants agreed to complete MIGS-compliant genome reports.

In brief, based on workshop discussions the GSC has developed the following ten-point Roadmap.

(1) *Update MIGS to version 1.1 before genome reports are accepted and post to the website for further community consultation.* Now available on the web, this version is more streamlined and strongly typed for the sake of future validation (e.g., selection from a controlled vocabulary is now expected for most values).

(2) *Implement GCat identifiers*. The group agreed they should take the form *NNNNNN*_GCAT (where *N* is a number from 0 to 9) to avoid any confusion with INSDC accession numbers. In taking this step, the GSC has paved the way towards creation and adoption of a community infrastructure for supporting MIGS compliance.

(3) *Produce a production version of GCat ready to accept published genome reports. *This is available at http://gensc.sf.net.

(4) *Develop guidelines for the submission of genomes reports. *These guidelines will emphasize that genomes should be submitted first to the INSDC and that the GSC then recommends that INSDC refer authors to GCat.

(5) *Actively work to generate MIGS-compliant genome reports*. The first valid reports are in the catalogue and the GSC will work with curators at key institutions (EBI, NCBI, JGI, TIGR, Sanger Institute) to curate further reports.

(6) *Build a batch upload facility into GCat*. This is required to allow GCat to deal with EMBL- and NCBI-produced lists of information about their genomes for the sake of populating GCat with content, quantifying the amount of optional information already associated with these genomes (e.g., the lat_lon qualifier) and working towards producing a single, global list of genomes in the public domain. Likewise, it will be necessary to support the future submission of Rob Edward's complete set of metagenomic datasets and, subsequent to the meeting, the full set of Sanger Institute genomes volunteered by Nick Thomson.

(7) *Develop a policy on ownership of the contents of the genome reports*. It was agreed that all data should be placed into the public domain as soon as deposited, and that a system of curation by the GSC and social tagging by any member of the community should be developed. Tatiana Tatusova (NCBI) stated that all data *must* be completely open access for NCBI's participation.

(8) *Seek funding to help support the implementation and adoption of MIGS*. Critical to the future success of this initiative will be the capacity of the GSC to find resources for curation (e.g., aid submitters, validate submissions, work with the INSDC, make sure information content stays compatible with changes in the MIGS specification and the introduction of new controlled vocabulary terms/ontologies). Although curation-based activities are difficult to fund, they are vital for the success of the project, and to the community as a whole. The cost of data curation by individual researchers is difficult to estimate, but considerably higher, both in financial and productivity terms. There is a pressing need for community to work with vetted datasets. Matt Kane (National Science Foundation) spoke briefly on the Research Coordination Network program offered by NSF which could be an opportunity to pursue for further support of networking, workshops, and related activities. The GSC agreed to explore this option further.

(9) *Return to NIEeS in 2007 for a 4th workshop*. Ideally, all of the above activities would be completed or well under way at the time of the 4th workshop and the GSC would be able to extend its Roadmap accordingly.

(10) *Complete MIGS 2.0*. We plan to complete MIGS 2.0, a production version of MIGS complete with an appropriate set of terms formalized within OBI [12] and other relevant ontologies, and a significantly improved version of the Genome Catalogue, by October 2007.

In conclusion, this workshop has produced an improved version of MIGS (v1.1), an updated XML schema (v1.1), consensus on a wide range of features to add to GCat, and further actions within the group that support the generation and submission of MIGS-compliant genome reports to GCat. Since the workshop, GCat identifiers have been implemented and the first MIGS-compliant genome reports for published and unpublished projects have been submitted [13–16]. A variety of value-added features have also been developed within the Genome Catalogue including the ability to view genomes on a map based on their latitude and longitude and the ability to access information using REST style web services. If the GSC can meet its target of producing the infrastructure required to support MIGS (specification, a working repository, and access to appropriate terms) it should put the community in a stronger position to push for enforcement of compliance. The GSC continues to make its open call for support and involvement in this initiative. The GSC welcomes new members, links to new projects, and researchers willing to describe the genomes with the submission of MIGS-compliant genome reports as part of the further development of this project. Anyone interested in knowing more about or joining this effort is encouraged to contact any of the coordinators or join the GSC mailing lists (http://gensc.sf.net).
